# Building a Robust 21st Century Chemical Testing Program at the U.S. Environmental Protection Agency: Recommendations for Strengthening Scientific Engagement

**DOI:** 10.1289/ehp.1408601

**Published:** 2014-10-24

**Authors:** Jennifer McPartland, Heather C. Dantzker, Christopher J. Portier

**Affiliations:** 1 Environmental Defense Fund, Washington, DC, USA; 2 Dantzker Consulting, Arlington, Virginia, USA

## Abstract

Background: Biological pathway-based chemical testing approaches are central to the National Research Council’s vision for 21st century toxicity testing. Approaches such as high-throughput *in vitro* screening offer the potential to evaluate thousands of chemicals faster and cheaper than ever before and to reduce testing on laboratory animals. Collaborative scientific engagement is important in addressing scientific issues arising in new federal chemical testing programs and for achieving stakeholder support of their use.

Objectives: We present two recommendations specifically focused on increasing scientific engagement in the U.S. Environmental Protection Agency (EPA) ToxCast™ initiative. Through these recommendations we seek to bolster the scientific foundation of federal chemical testing efforts such as ToxCast™ and the public health decisions that rely upon them.

Discussion: Environmental Defense Fund works across disciplines and with diverse groups to improve the science underlying environmental health decisions. We propose that the U.S. EPA can strengthen the scientific foundation of its new chemical testing efforts and increase support for them in the scientific research community by *a*) expanding and diversifying scientific input into the development and application of new chemical testing methods through collaborative workshops, and *b*) seeking out mutually beneficial research partnerships.

Conclusions: Our recommendations provide concrete actions for the U.S. EPA to increase and diversify engagement with the scientific research community in its ToxCast™ initiative. We believe that such engagement will help ensure that new chemical testing data are scientifically robust and that the U.S. EPA gains the support and acceptance needed to sustain new testing efforts to protect public health.

Citation: McPartland J, Dantzker HC, Portier CJ. 2015. Building a robust 21st century chemical testing program at the U.S. Environmental Protection Agency: recommendations for strengthening scientific engagement. Environ Health Perspect 123:1–5; http://dx.doi.org/10.1289/ehp.1408601

## Introduction

There is a significant lack of health and safety information on most chemicals in the marketplace. The U.S. Environmental Protection Agency’s (EPA) chemical inventory includes nearly 85,000 chemicals available for use in the United States (U.S. EPA 2014j), yet only a small subset of these chemicals has been sufficiently characterized in order to draw conclusions about the chemicals’ toxicity for regulatory decision making (Judson et al. 2009; Landrigan and Goldman 2014). This lack of data is largely the result of inadequate chemical safety laws (Denison 2009; Landrigan and Goldman 2014)—notably the Toxic Substances Control Act (TSCA), which places onerous administrative and evidentiary burdens on government to require chemical testing—combined with the resource needs and time required to evaluate chemicals in traditional whole animal studies [National Research Council (NRC) 2007]. Furthermore, regulatory or guideline test protocols are limited in their ability to fully evaluate toxicity at various life stages, examine unique susceptibilities among vulnerable populations, efficiently assess mixtures, and provide mechanistic insight into chemical toxicity (NRC 2007; Portier 2009).

Advances in molecular biology and computational science as well as greater insight into the mechanisms of chemical toxicity, disorders, and disease (Portier 2001) have created the opportunity to greatly expand the number of chemicals evaluated for biological effects. The U.S. EPA, the National Institute of Environmental Health Sciences (NIEHS), the National Toxicology Program (NTP), the National Institutes of Health (NIH), the Food and Drug Administration (FDA), the Agency for Toxic Substances and Disease Registry (ATSDR), and others have been working for more than a decade to advance new chemical testing approaches that are rapid, use fewer resources and animals, and at a minimum retain—but ideally exceed—the predictive power of current testing methods, while also offering the potential to gain additional biological insight (Bucher 2013; U.S. EPA 2014d, 2014g). Key among these technologies is high-throughput (HT) *in vitro* testing, which is designed to screen thousands of chemicals over spans of days or weeks—a quantum leap over traditional animal tests that can take months or years to complete. HT *in vitro* testing forms the basis of two major federal chemical testing efforts, the National Center for Computational Toxicology (NCCT) ToxCast™ initiative (U.S. EPA 2014d) and the Tox21 partnership between the U.S. EPA, the NIH (NIEHS/NTP and the National Center for Advancing Translational Sciences), and the FDA (Tice et al. 2013; U.S. EPA 2014e). These initiatives have generated data on thousands of chemicals using an array of new assays across multiple *in vitro* testing platforms (Tice et al. 2013; U.S. EPA 2014d, 2014h). Using computational tools, research groups are already beginning to use these data to characterize chemical hazard and risk (e.g., Sedykh et al. 2011; Sirenko et al. 2014; Wetmore et al. 2013). Given the dearth of toxicity information on chemicals, HT methods hold great potential to fill data gaps and expand our understanding of the potential health and environmental effects of chemicals. Importantly, however, health-protective application of these methods ultimately depends on the scientific integrity of the assays, the scientifically sound interpretation of the data emerging from them, and the transparency of the entire process.

In 2004 the NTP published a seminal vision and roadmap in recognition and anticipation of this emerging revolution in chemical testing (NTP 2004a, 2004b). Building on this foundation, the NRC published a report that outlined and established support for a gradual paradigm shift in toxicity testing (NRC 2007). A central feature of these publications is a reduction in traditional toxicity testing approaches that rely on whole animals to examine apical end points (e.g., mortality, tumors, reproductive and developmental effects) and a shift to more predictive approaches using assays that measure perturbations of biological pathways. From method development and validation to implementation and institutional transitions, actualizing this shift is unquestionably challenging, especially within the confines of limited resources. Appreciating the significance of this undertaking, both the NTP and the NRC emphasized the important role of the broader scientific community in developing new testing strategies. Indeed, the NRC report (NRC 2007) stated that

[a]cceptance of the committee’s vision in the scientific community will require further elaboration of the technical details of its implementation and generation of new scientific evidence to support the move away from apical end points to perturbations of toxicity pathways. The broad participation of the scientific community in the elaboration of the committee’s vision for toxicity testing is essential for its success.

The ultimate objective of any improvement in toxicity testing is the protection of public health and the environment. Broadly speaking, there are three basic goals that must be achieved if a new chemical testing paradigm is to be realized: *a*) development of a scientific foundation that supports understanding of the genesis and progression of environmentally influenced disease; *b*) development and scientific acceptance of a testing and analysis paradigm built on this foundation; and *c*) policies that encourage the use of this new knowledge to reduce exposures to harmful chemicals in the environment. Such an outcome can emerge only if a diverse group of stakeholders is involved at all steps of a flexible process that fosters incorporation of advances in scientific knowledge and technology.

In recent years, Environmental Defense Fund (EDF) has brought together stakeholders from government, academia, the public interest community, and industry to discuss perspectives on new federal chemical testing programs. We believe that stakeholder engagement, especially from the scientific research community, is needed for success in the development, scientific acceptance, and application of a new chemical testing paradigm. In addition to activities we have initiated, EDF is an active participant in the U.S. EPA’s Communities of Practice, ToxCast™ stakeholder meetings, as well as other conversations around new chemical-testing efforts. Building on our experience, here we present two specific, actionable recommendations focused on increasing engagement of the broader scientific research community in the U.S. EPA’s ToxCast™ initiative.

Our first recommendation is to expand the scientific conversation around—and input into—the U.S. EPA’s ToxCast™ initiative through a series of collaborative workshops. Our second recommendation is to expand collaborations between the U.S. EPA’s ToxCast™ initiative and the research community through mutually beneficial research partnerships. EDF appreciates the challenges that regulatory agencies such as the U.S. EPA face in meeting statutory obligations to ensure chemical safety, given the insufficient information available and the paucity of resources to fill data gaps. Our intent here is not to complicate these responsibilities but rather to bolster the ability of the U.S. EPA to use data emerging from ToxCast™, Tox21, and similar initiatives. We believe that these modest steps to more actively engage the broader scientific research community, taken now, will facilitate incorporation of the best available scientific understanding of biological systems into the development and application of emerging HT methods. This, in turn, should lead to broader support and acceptance for applying them in regulatory chemical testing programs and related decision making.

Our recommendations for near-term U.S. EPA engagement with the scientific research community go beyond engagement with the traditional stakeholders with an interest in regulatory chemical testing to include those researchers within the environmental health sciences (EHS) field who have deep understanding of the basic mechanisms by which environmental agents can affect normal biological function. From our experience, there is low engagement of the EHS community with ToxCast™ and the corresponding data now available. We believe that the U.S. EPA must engage EHS researchers more directly to help strengthen the foundation of its chemical testing efforts. Active engagement should entail the U.S. EPA connecting with EHS researchers in their own settings, for example, by the U.S. EPA participating in the conferences of medical and disease-focused professional societies. We anticipate that by the U.S. EPA working directly with EHS researchers, over time interest and collaboration would expand to new researchers focused on elucidating the etiology and pathogenesis of disorders and diseases, including those who have not conventionally examined environmental contributions. This bridging approach would provide the U.S. EPA with a practical path forward for increasing the diversity of scientific input into the ToxCast™ initiative while affording opportunities for the agency to share its perspectives, constraints, and regulatory demands with the scientific research community. This collaborative engagement will help grow the broad, diverse base of scientific support that will be needed to fully realize the paradigm shift in toxicity testing.

## Discussion

We agree with the NRC’s view that “toxicity testing is useful ultimately only if it can be used to facilitate more informed and efficient responses to the public health concerns of regulators, industry, and the public” (NRC 2007). The focus of the U.S. EPA’s research efforts on application, as opposed to more basic research, reflects the practical considerations arising from the U.S. EPA’s statutory mandates, guidance development, and related decision-making needs. Yet whether data are used to evaluate new or existing environmental agents, contaminated sites, or potential environmental contributors to disease, or to compare relative risks (NRC 2007), acceptance of the scientific foundation for all of these efforts remains critical and is strengthened by a robust engagement of the broader scientific community.

Integrating new testing approaches into environmental health assessments presents challenges, including grappling with new data types, standardizing methods, validating predictions, and addressing uncertainty. Acceptance of any new testing paradigm will require that stakeholders and the larger public are confident that decisions, even in situations of incomplete information or uncertainty, are based on the best available scientific understanding and are transparent (NRC 2008). To address such challenges, one of the NRC’s five main recommendations for successful assessments and decisions was that those issues with “substantial scientific content should be supported with collaborative, broadly based, integrated, and iterative analytic-deliberative processes” (NRC 2008). Gleaned from empirical research, these recommendations reflect decades of valuable experience integrating science and decision making that should be applied to the U.S. EPA’s ToxCast™ initiative. To ensure that chemical testing assays and prediction models are recognized as scientifically rigorous and robust, those with expertise relevant to the development and interpretation of those approaches need to be included in the process. Fostering collaborative engagement will engender advancement of our understanding of chemical effects through scientific discovery, innovation, and capacity building.

*Recommendation 1. Collaborative workshops: expand the conversation and increase the diversity of scientific input.* Our first recommendation for broadening engagement in the U.S. EPA’s ToxCast™ initiative is to initiate a series of workshops that engage agency and outside researchers in collaborative inquiry and problem solving in a more explicit and substantive way than has occurred to date. For example, a workshop could *a*) broadly examine currently understood biological mechanisms underlying the etiology and pathogenesis of a particular disorder or disease (e.g., asthma, Parkinson’s, thyroid toxicity); *b*) evaluate known environmental contributors to the particular disorder or disease in light of these mechanisms; and *c*) assess data from the current ToxCast™ and Tox21 testing batteries and prediction models in relationship to these mechanistic and environmental insights.

The 2011 NTP workshop “Role of Environmental Chemicals in Diabetes and Obesity” serves as a useful model for the development of this type of workshop (Thayer et al. 2012). The purpose of the workshop was to review the state of the science regarding the contribution of certain chemical exposures to diabetes and obesity as part of a larger effort to develop a research agenda on this issue. Scientific experts across diverse fields were asked to critically examine pertinent human and experimental animal literature and provide an assessment of the strength and biological plausibility of findings, the most useful and relevant experimental models, and data gaps for future research. Data from ToxCast™ were shared with participants to aid in evaluation of biological plausibility, identify chemicals that interact with relevant mechanistic targets but have not yet been studied for effects related to diabetes and obesity, and recommend additional mechanistic targets for inclusion in the HT testing battery. The highly interactive, collaborative format of the workshop afforded an opportunity for the NTP to explain ToxCast™ to researchers and to receive real-time expert analysis and feedback on the ToxCast™ data. After the meeting, the NTP worked with select participants to use ToxCast™ data to identify chemicals of interest for further evaluation in their own laboratories, yielding another valuable review of the HT data for the agency.

A 2007 workshop, “Moving Upstream: A Workshop on Evaluating Adverse Upstream Endpoints for Improved Decision Making and Risk Assessment,” brought together academic and government scientists to review relationships between chemical exposures, early biological effects, and later overt effects (Woodruff et al. 2008). Using three prepared case studies on thyroid hormone disruption, antiandrogen effects, and immune system disruption, participants examined what is known in each case about precursor effects, their relationship to downstream effects, challenges to the use of precursor effects in risk assessment, and what additional data are needed to better understand these relationships (Parham et al. 2012; Wise et al. 2012; Woodruff et al. 2008). The U.S. EPA’s ToxCast™ initiative was in its infancy at the time of that meeting. It would be worthwhile to hold a similar workshop now and include an evaluation of ToxCast™ and Tox21 data as part of the agenda.

Like the meetings described here, the workshops we envision would provide a mechanism for scientific input into the U.S. EPA’s ToxCast™ initiative through collaboration between external researchers and agency scientists. Such a mechanism does not currently exist and is needed to ensure that development of new regulatory testing efforts move forward in a way that receives broad scientific support and acceptance.

*Recommendation 2. Creative collaborations: seek out mutually beneficial research partnerships.* Currently, the U.S. EPA’s ToxCast™ initiative works with other federal and state agencies, industry and trade associations, universities, and other research and nongovernmental organizations. These relationships are formalized through memoranda of understanding, material transfer agreements, cooperative research and development agreements, and through research grants (U.S. EPA 2014b). Since 2004, the U.S. EPA has awarded > $40 million in extramural research funding related to computational toxicology as part of its Science to Achieve Results (STAR) grant program (U.S. EPA 2012, 2014c, 2014f). The U.S. EPA has also recently launched crowd-sourcing challenges that focus on the development of algorithms to predict toxicological end points using ToxCast™ data (U.S. EPA 2014h). The U.S. EPA hosts meetings and other forums to showcase advancements in their program [e.g., U.S. EPA’s Communities of Practice, (U.S. EPA 2014a); ToxCast™ Data Analysis Summit (U.S. EPA 2014i)]. Although these efforts are valuable, they are limited in their ability to reach EHS and other investigators with the knowledge and expertise needed to expand and strengthen the underlying scientific foundation of ToxCast™ and to foster substantive, scientific discussions with U.S. EPA scientists to address critical scientific issues at hand. Different collaborative partnerships are needed to attract and facilitate discussions between agency and outside researchers.

A key challenge is finding ways to solicit expertise from the EHS research community in a manner that is beneficial both to the U.S. EPA and to external researchers. We recommend that the U.S. EPA work with the NIEHS and other federal agencies to establish and incentivize mutually beneficial research partnerships with the EHS research community. For example, small add-on grants to researchers already funded by the NIEHS and others would immediately broaden the research base for the U.S. EPA’s ToxCast™ initiative and expose more scientists to the scientific challenges involved. In turn, researchers could identify opportunities for improvement, such as adding new biological targets to the ToxCast™ testing battery, eliminating underperforming assays, and modifying toxicity prediction models built using ToxCast™ data. The U.S. EPA appears interested and open to this type of input. The U.S. EPA will need to put mechanisms in place to communicate, evaluate, and integrate new scientific findings into its testing programs. Two current collaborations reflect the type of research partnerships we envision.

Investigating potential environmental chemical contributors to very early onset inflammatory bowel disease in children using ToxCast™ and Tox21 HT *in vitro* data. In 2012, Harvard clinicians and researchers partnered with U.S. EPA scientists to explore potential environmental contributors to very early onset inflammatory bowel disease (VEO-IBD) in children. The overall program led by Scott Snapper (Children’s Hospital, Harvard Medical School) and titled “Very Early Onset Inflammatory Bowel Disease” encompasses three projects. One of these focuses on identifying environmental factors affecting VEO-IBD pathogenesis and is co-led by Russ Hauser (Harvard School of Public Health) and Francisco Quintana and Joshua Korzenik (both of Brigham and Women’s Hospital and Harvard Medical School). The proposal to use ToxCast™ data in Hauser’s ongoing IBD research was submitted to and funded by the Helmsley Foundation via Harvard University’s Catalyst program and the Harvard Institute of Translational Immunology (Hauser R, personal communication).

Harvard IBD experts reviewed the list of biological targets represented in the ToxCast™ battery of assays to identify those assays with greatest potential relevance to VEO-IBD. Of the approximately 500 assays reviewed, about two dozen assays were selected as high priority. It is important to note that not all of the Harvard researchers’ biological targets of interest were represented in the available HT assays. U.S. EPA scientists subsequently developed an activity heat map of almost 1,000 tested substances (e.g., industrial chemicals, dietary compounds, pharmaceuticals) across the high-priority assays. The Harvard group identified approximately 40 substances of greatest interest using the heat map analysis. These 40 substances will be tested in zebrafish models of colitis in Quintana’s laboratory to further investigate the potential relationship between chemical exposure and early-onset IBD. The results of these zebrafish studies will be complemented with analyses performed on human immune cells *in vitro* and used to prioritize substances for potential future human epidemiological studies (Hauser R, personal communication).

This collaboration is of considerable value to the U.S. EPA and Harvard University scientists and clinicians. The research partnership will provide the U.S. EPA with expert analysis of the relevance of the biological targets represented in ToxCast™ assays to VEO-IBD, as well as insight into additional VEO-IBD targets missing from the ToxCast™ initiative. Planned research from higher-order testing in zebrafish and human studies will provide additional information on the relevance of the ToxCast™ assays. The collaboration has established new relationships and broadened the scientific networks of both U.S. EPA and basic research scientists. For the Harvard investigators, the effort supports ongoing research and offers the potential for breakthroughs in understanding environmental contributors to VEO-IBD. It also enables researchers and clinicians with little or no experience with environmental risk factors to interact with agency scientists and broaden the scope of their work. Importantly, it provides a model—in line with the NTP’s and NRC’s visions—for using predictive toxicological modeling to investigate the potential role of environmental chemicals in the etiology of complex diseases.

Chemicals and Breast Cancer: Building on National Initiatives for Chemical Safety Screening. Another notable research project, Chemicals and Breast Cancer: Building on National Initiatives for Chemical Safety Screening, focuses on the development of rapid *in vitro* screens for breast carcinogens in mammary cells [California Breast Cancer Research Program (CBCRP) 2014]. This research project, funded by the CBCRP Special Research Initiatives (SRI), is led by Chris Vulpe at the University of California, Berkeley, and includes as partners the Lawrence Berkeley National Laboratory and the Silent Spring Institute, a nonprofit scientific research center that studies environmental influences on women’s health. The goal of the project is to identify ToxCast™ and Tox21 HT assays that can discriminate among three types of chemicals: rodent mammary gland carcinogens, chemicals that alter mammary gland development in rodents, and chemicals that did not induce tumors in NTP rodent bioassays. The research team drew upon the expertise of breast cancer biologists and consulted with the U.S. EPA to identify the assays most relevant to breast carcinogenesis on the basis of their predictive power for developmental and reproductive toxicity and endocrine activity. These select assays will be transferred into breast cell lines and more complex breast cell models for testing of > 100 chemicals. In addition, the SRI research group is developing assays to measure expression of certain breast cancer–related genes, new end points not currently evaluated by ToxCast™.

Like the Harvard IBD collaboration, this project uses new experimental and computational tools to examine potential chemical contributors to disease and provides mutual benefits for both the extramural researchers and federal agencies. The U.S. EPA and the NTP/NIEHS are providing SRI researchers with test substances, access to and guidance on the interpretation of the ToxCast™ and Tox21 data, and advice on experimental design. In turn, the SRI researchers are providing the U.S. EPA and other federal partners additional review of selected assays and input on new assays needed to cover biological space not yet represented in the current ToxCast™ and Tox21 assays. As a result of this work, the SRI partners have already identified areas for future investigation (Rudel R, personal communication).

Opportunities for expanding engagement. From the EDF’s perspective, the two research projects described above are models for increasing the contributions of the larger scientific research community to the U.S. EPA’s ToxCast™ initiative. Leveraging existing resources, these partnerships enable new lines of scientific inquiry while strengthening the scientific basis of both ToxCast™ and Tox21. Shared perspectives, new knowledge, and greater understanding are the result. Although the partnerships we described here are to some degree the result of serendipity, we encourage the U.S. EPA to more proactively seek out these types of opportunities. Specifically, the U.S. EPA should consider

Identifying existing U.S. EPA grant recipients who would likely be interested in using ToxCast™ or Tox21 data or initiating new projects in this area
Presenting at scientific professional society meetings beyond those that are specifically focused on toxicology (e.g., Endocrine Society, Society for Neuroscience, American College of Epidemiology)
Initiating conversations with NIEHS-funded extramural researchers and research centers (e.g., NIEHS Centers for Children’s Environmental Health and Disease Prevention Research) to explore how ToxCast™ and Tox21 data could be used in their investigations.

## Conclusions

Decisions regarding environmental health risks include both science and broader public discourse on that science. Although public discourse can be acrimonious at times, successful dialogue and decision making are improved only with broad scientific engagement. There are real benefits and opportunities for both federal agencies and the broader scientific research community to collaborate in the establishment of a robust scientific foundation for 21st century chemical testing. Collaborative engagement is important to ensure that diverse, and in some cases novel, scientific knowledge and perspectives are considered in the development of new chemical testing approaches. Indeed, the very foundation of the vision for 21st century chemical testing originates from basic research that investigates and characterizes biological mechanisms underlying various health disorders and diseases. Application of this same biological understanding has resulted in major drug discoveries and improvements in health, and it can and should similarly form the backbone of health-protective, cutting-edge chemical testing efforts, particularly given the anticipated increasing role that these new approaches will play in public health decision making.

We believe that the best way to ensure that the most current scientific understanding is incorporated into chemical testing frameworks, tools, and prediction models is to engage research scientists in their development, review, and application. Although this notion is perhaps obvious, its execution will take considerable effort and creative approaches. Such substantive scientific engagement will also be critical for securing public acceptance of regulatory activities that rely on new chemical testing data. Determining how best to effectively and productively involve the scientific research community will be an iterative process. EDF looks forward to helping catalyze and support this effort through its role as a public and scientific stakeholder committed to protecting human and environmental health from harmful chemical exposures.
